# Expanding HIV testing and linkage to care in southwestern Uganda with community health extension workers

**DOI:** 10.7448/IAS.20.5.21633

**Published:** 2017-07-21

**Authors:** Stephen Asiimwe, Jennifer M. Ross, Anthony Arinaitwe, Obed Tumusiime, Bosco Turyamureeba, D. Allen Roberts, Gabrielle O’Malley, Ruanne V. Barnabas

**Affiliations:** ^a^ Integrated Community Based Initiatives, Kabwohe, Uganda; ^b^ Division of Allergy and Infectious Disease, University of Washington, Seattle, WA, USA; ^c^ School of Medicine, University of Washington, Seattle, WA, USA; ^d^ Department of Global Health, University of Washington, Seattle, WA, USA; ^e^ Departments of Global Health, Medicine (Allergy and Infectious Disease), and Epidemiology, University of Washington, Seattle, WA, USA

**Keywords:** HIV, community health workers, Uganda, task shifting, linkage to care, scalability

## Abstract

**Introduction**: Achieving the UNAIDS goals of 90–90-90 will require more than doubling the number of people accessing HIV care in Uganda. Community-based programmes for entry into HIV care are effective strategies to expand access to HIV care, but few programmes have been evaluated with a particular focus on scale-up.

**Methods**: Integrated Community Based Initiatives, a Uganda-based non-governmental organization, designed and implemented a programme of community-based HIV counselling and testing and facilitated linkage to care utilizing community health extension workers (CHEWs) in rural Sheema District, Uganda. CHEWs performed programme activities during 1 October 2015 through 31 March 2016. Outcomes for this evaluation were (1) the number of people tested for HIV, and (2) the proportion of those testing positive who were seen at an ART clinic within three months of their positive test, and (3) the cost of the programme per person newly diagnosed with HIV. Microcosting methods were used to calculate the programme costs. Program scalability factors were evaluated using a published framework.

**Results**: Sixty-two CHEWs attended a five-day training that introduced the biology of HIV, the conduct of confidential HIV testing, HIV prevention messages, and linkage, referral, and reporting requirements. CHEWs received a $30 monthly stipend and a field testing kit that included a bicycle, field bag, umbrella, gumboots, reporting booklet, pens, and HIV testing materials. Trained CHEWs tested 43,696 persons for HIV infection during the six-month programme period. Nine-hundred seventy-four participants (2.2%) were identified as HIV positive, and 623 participants (64%) were linked to HIV care. An estimated 69% of adult residents received testing as part of this campaign. The programme cost $3.02 per person test, $135.70 per positive person identified, and $212.15 per HIV-positive person linked to care.

**Conclusions**: Lay community health extension workers (CHEWs) can be rapidly trained to scale-up home-based HIV testing and counselling (HTC) and linkage to care in a high-quality and low-cost manner to large numbers of people in a rural, high burden setting. A combination HIV testing approach, such as adding partner testing to community-based testing, could increase the proportion of HIV-positive persons identified.

## Introduction

Achieving the UNAIDS targets of identifying 90% of HIV-positive persons, initiating antiretroviral therapy (ART) among 90% of HIV-positive persons, and achieving 90% viral suppression among those on ART in Uganda by 2020 requires ART access for an additional 800,000 people, roughly doubling the current programme size [1,2]. Government per capita spending on health is an estimated $9 US dollars per person annually in Uganda, and the majority of the HIV programme is funded through international donors [3,4]. Meeting the need for programme scale-up in Uganda will require expansion of cost-effective HIV services beyond public sector health facilities. Community-based entry into HIV care and task-shifting of care tasks are effective and cost-effective strategies to expand access to HIV care [5–8]. Furthermore, community-based strategies, such as mobile testing, reach men, who are less likely than women to receive HIV testing at a health facility (42% men versus 58% women) [7]. Key informant interviews conducted recently among Ugandan public health stakeholders identified expansion of home-based HIV care services as a key measure to improve the delivery of HIV services [9]. As community-based strategies form part of Uganda’s national AIDS policy [2], their implementation must be carefully evaluated and described in the literature to inform future efforts.

Interventions successfully implemented in research settings may prove less successful once they move out of the hands of researchers and into the community. Therefore, process and outcome evaluations of these health interventions implemented in real world settings are important for informing decisions of whether and how to take to scale interventions proven efficacious in the controlled environment of a research study. We adapted a scalability framework developed by Milat and colleagues [10,11] to assess the implementation and effectiveness of a home based programme for HIV testing, counselling, and linkage to care programme implemented in a high-burden area of rural Uganda. Results may help other community-based HIV care providers adapt similar programmes to their own context.

## Methods

### Programme setting

**Figure 1.**
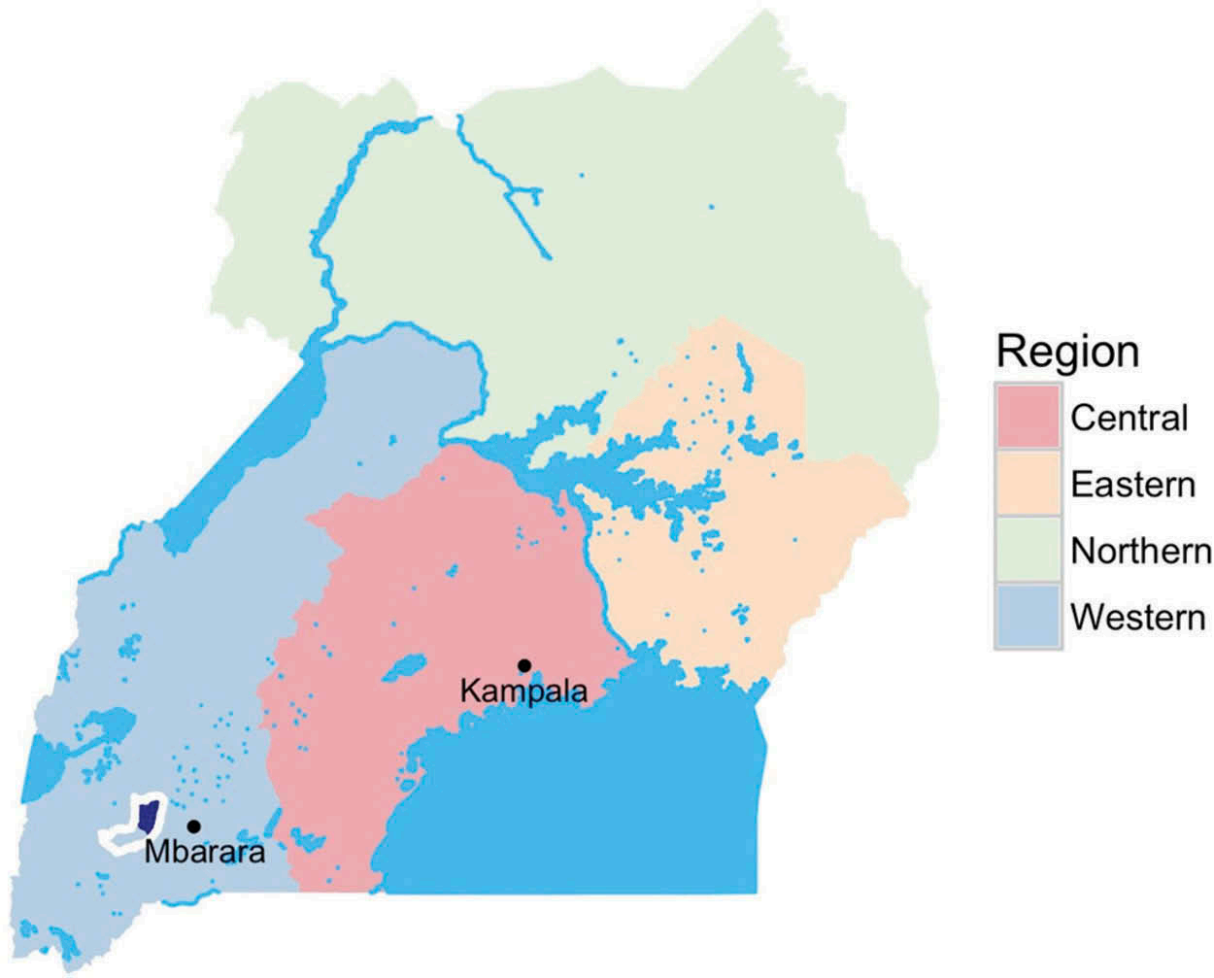
Map of Uganda showing location of programme area. Map of Uganda showing Sheema District outlined in white with programme location in northern sub-counties of Sheema District shown in dark blue.

In 2015, the Uganda Ministry of Health and UNAIDS supported Integrated Community Based Initiatives (ICOBI), a Uganda-based non-governmental organization, to design and implement a programme of community-based HIV counselling and testing and facilitated linkage to care utilizing community health extension workers (CHEWs) in Sheema District, Uganda, which has an estimated HIV prevalence between 4% and 5% [12,13]. The testing programme adapted a model that had been used in clinical trials of community-based HIV testing and counselling and linkage [14] for use in this community-based context without research staff. This programme took place in six rural sub-counties of Sheema District in southwestern Uganda (Figure 1) between 1 September 2015 and 30 April 2016, with all testing completed during a six month period between October and March. Approximately 126,000 persons live in the study area. The intervention targeted all adults ages 15–64 years living in the study area.

### Procedure for home-based testing

At each household, CHEWs obtained verbal informed consent and then offered HIV counselling and rapid testing using the nationally approved algorithm of Determine® for screening, StatPak® for confirmation and Unigold® as tie breaker to each adult greater than age 15. CHEWs collected blood samples from every person with a positive rapid HIV test for confirmatory ELISA testing at Kabwohe Clinical Research Center (KCRC) reference laboratory. Blood samples were also collected from every 10th person with a negative rapid HIV test for quality assurance testing at KCRC. CHEWs delivered an HIV prevention education message to every person tested.

### Facilitation of linkage to care and assessment of linkage

After post-test counselling, a referral slip was provided to every HIV positive person identified. The slip had the details of the test results as well as the reason for referral and a contact person at the health care facility. Each of the health facilities offering HIV/ART services in the programme area have linkage coordinators (health centre liaisons) who assist clients entering HIV care. Participants who accessed care at the health facilities were provided with written documentation of their visit. CHEWs returned to the homes of HBCT participants and asked to see clinic documentation to follow-up on whether participants had successfully linked to care.

### Data management and statistical analysis

CHEWs delivered notebooks documenting the outcome of rapid tests to ICOBI data managers monthly. Data were entered into a password-protected Microsoft Access database. Mean values were calculated for continuous variables (age) and proportions for categorical variables (gender, marital status, educational attainment, prior testing history, and linkage to care). Data analysis was performed using R version 3.2.3 [15].

### Coverage

Intervention coverage among the adult population (15–64 years) was estimated using the national population age structure from the 2014 census [16], stratified by urban and rural locations. To estimate the number of people who would decline a HIV test due to a previous HIV diagnosis, the HIV prevalence of Sheema District was multiplied by an estimate of the percentage of people living with HIV who had previously received a positive HIV test (39.6%, 2011 AIDS Indicator Survey) [17]. This number was excluded from the denominator for intervention coverage. This approach assumes that those tested in the intervention reside in Sheema North.

### Evaluation

The primary outcomes to evaluate the effectiveness of the testing programme were (1) the number of HIV tests performed, (2) the proportion of positive persons who were seen at an ART clinic within three months of their positive test, and (3) the cost of the programme per person newly diagnosed with HIV. These outcomes were compared with those obtained by all of the public sector health facilities in the same catchment area of the programme between 1 October 2015 and 31 March 2016. Health facility data included the number of HIV tests performed, and the proportion of positive persons seen at an ART clinic within three months of their positive test. Outcomes obtained by public health centre facilities were provided by the biostatistician at the Sheema District Health Office.

Program implementation and outcome evaluation results were then assessed against the scalability considerations framework developed by Milat and colleagues [10,11]. These considerations include effectiveness, workforce and organizational needs, cost considerations, intervention delivery feasibility, contextual factors, and appropriate evaluation approaches ICOBI collaborated with investigators at the University of Washington for the programme evaluation and scalability assessment.

### Microcosting

Following the methods of previous costing studies, a microcosting analysis was conducted taking a programmatic perspective [6,18,19]. Costs were estimated from budget, expenditure sheets, and staff interviews. Mutually exclusive cost categories include personnel, transportation, equipment, supplies, building and overhead, and start-up. Capital costs were assumed to have a five-year useful life span discounted annually at 3% [20,21], while training and mobilization were assumed to recur annually. Costs were inflated to 2016 US dollars (USD) using Uganda consumer price indices.

### Human subjects protection

This analysis involved evaluation of routine programme data collected and aggregated without any personal identifiers. It did not require informed consent or human subjects review.

## Results

### Workforce

Staff of the Uganda-based non-governmental organization Integrated Community Based Initiatives (ICOBI) implemented the programme. ICOBI staff served as programme directors, coordinators, monitoring and evaluation specialists, and laboratory and data managers. ICOBI programme staff recruited two CHEWs from each of the 31 administrative parishes in the study area. All persons targeted for recruitment had previously received ministry of health training in community health work as a Village Health Team member. District Health Officers and ICOBI identified potential CHEWs for recruitment through written and oral interviews that assessed their basic knowledge of HIV as well as their ability to express themselves within the community.

**Figure 2.**
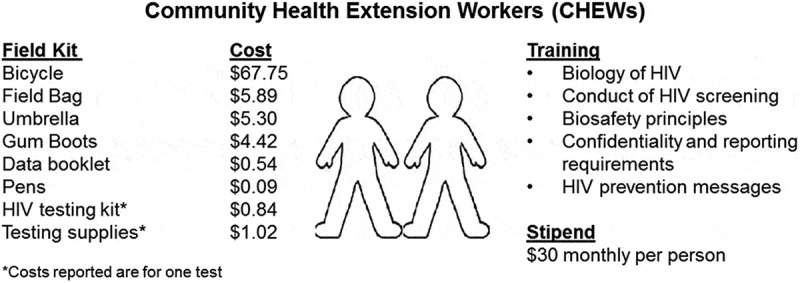
Training and equipment provided to community health extension workers.

CHEWs attended a five-day training that introduced the biology of HIV, the conduct of HIV screening test, the maintenance of confidentiality regarding HIV testing, HIV prevention messages, principles of biosafety, and linkage, referral, and reporting requirements (Figure 2). The training curriculum followed Uganda Ministry of Health AIDS Control Program HIV/AIDS awareness training materials. CHEWs were each provided a $30 monthly stipend and a field testing kit that included a bicycle, field bag, umbrella, gumboots, reporting booklet, pens, HIV testing kits, biohazard bags, lancets and gloves. Supplies were replenished monthly. At the initiation of the project, ICOBI staff assisted each pair of CHEWs to develop a route for approaching each consecutive household in their administrative parish.

### Intervention delivery

ICOBI staff designed the intervention programme in collaboration with local health stakeholders. Project staff hosted a launch function at the start of the intervention to introduce the project to community health stakeholders. The proportion of CHEWs submitting a monthly testing record was highest in the first month (100%), lowest in the second month (79%) and had a mean monthly value of 91% over the six months of the programme.

### Effectiveness and reach

CHEWs conducted home based HIV counselling and testing for 43,696 participants during the six months of the programme (Table 1). Nine-hundred seventy-four persons (2.2%) were identified as HIV positive, and 623 persons (64%) were linked to HIV care at community health care facilities. The remaining 351 persons (36%) were not linked to care within three months of their positive test. In comparison, public sector health facilities in the same region tested 15,117 persons for HIV infection and recorded 778 (5.1%) positive tests over the same period of 1 October 2015 through 31 March 2016. Public sector health facilities linked 592 (76.1%) of HIV-positive persons to care, which was higher than the proportion linked by CHEWs (difference 12.1%, 95% CI 7.8%–16.5%, *p* < 0.001, binomial proportions test).

**Table 1.** Characteristics of participants contacted by community health extension workers (CHEWs) for HIV home-based counselling and testing (*N* = 43,696).

		Male	Female
		*n* = 20729 (47%)	*n* = 22967 (53%)
Age (years)	Mean (SD)	31 (13)	30 (13)
	<18	1693 (8%)	2293 (10%)
	18–24	5754 (28%)	7028 (31%)
	25–34	6377 (31%)	6550 (29%)
	35–44	3542 (17%)	3764 (16%)
	45+	3363 (16%)	3332 (15%)
Marital status^a^	Single	8759 (42%)	8346 (36%)
	Married/Cohabiting	11270 (54%)	12470 (54%)
	Divorced/Separated	434 (2%)	908 (4%)
	Widow/Widower	264 (1%)	1243 (5%)
Education level	Not Educated	930 (5%)	1396 (6%)
	Primary	11454 (55%)	12613 (55%)
	Secondary	7104 (34%)	7881 (34%)
	Tertiary	1241 (6%)	1077 (5%)
Tested previously^b^	Yes	16124 (78%)	18004 (78%)
	No	4521 (22%)	4868 (21%)
Test result	Negative	20325 (98.1%)	22397 (97.5%)
	Positive	404 (1.9%)	570 (2.5%)
Linked to care^c^	Yes	623 (64%)	
	No	351 (36%)	

^a^Missing for two persons. ^b^Missing for 179 persons (84 men and 95 women). ^c^Data not available by gender.

All 974 samples that were positive for HIV by a rapid test were also positive by confirmatory ELISA. None of the 934 negative samples selected for confirmatory testing were positive by ELISA.

**Figure 3.**
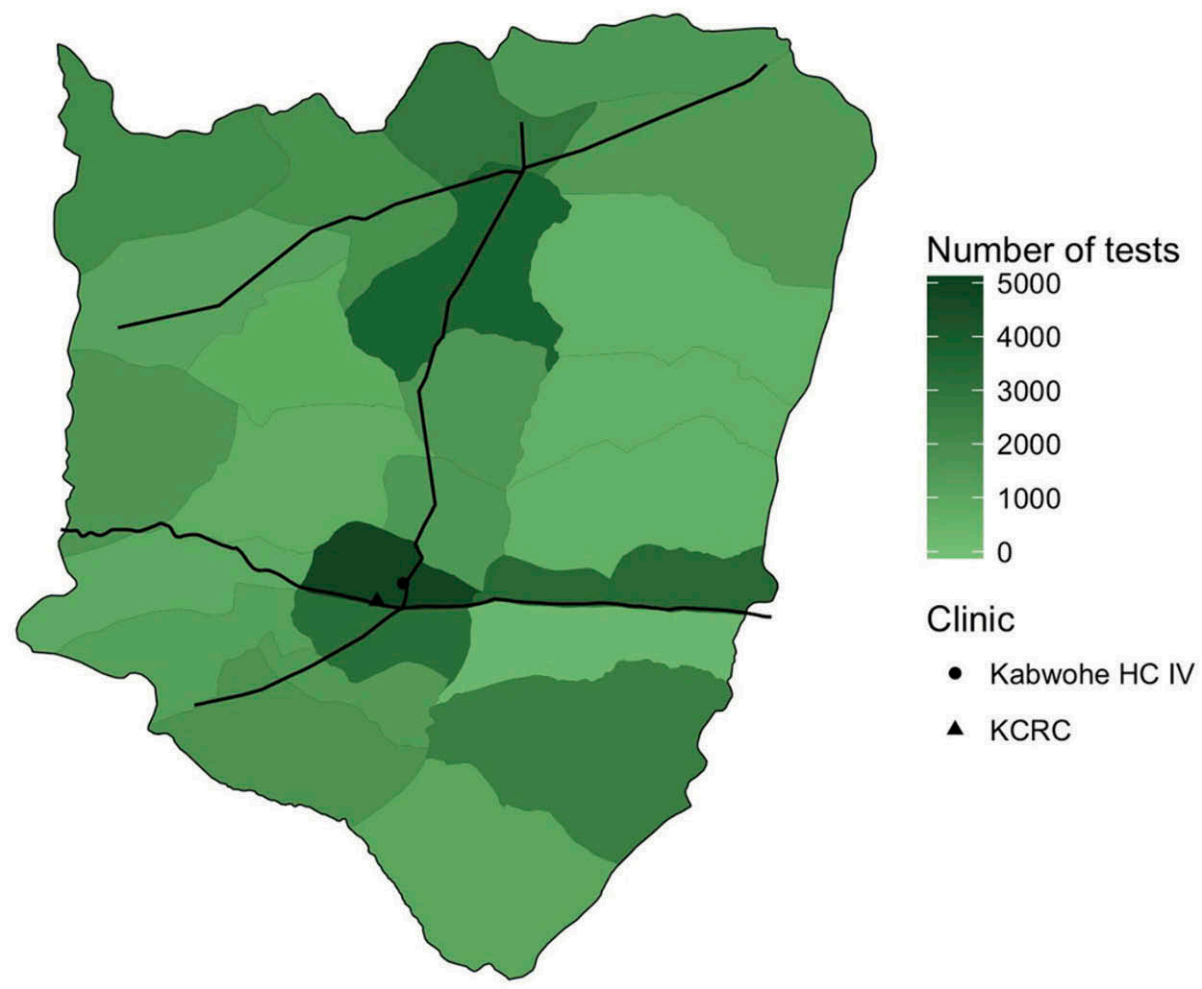
Map of northern parishes of Sheema District, Uganda indicating number of HIV tests conducted in each parish by CHEWs during the intervention period. Black lines indicate major roads. Kabwohe level IV health centre and Kabwohe Clinical Research Center also shown.

CHEWs conducted home based testing and counselling for an estimated 69.4% of the adult residents of northern Sheema District. The parish with the greatest number of tests performed was Kabwohe Itendero Town Council, which is also where the Kabwohe Clinical Research Centre is located (Figure 3).

### Cost considerations

**Figure 4.**
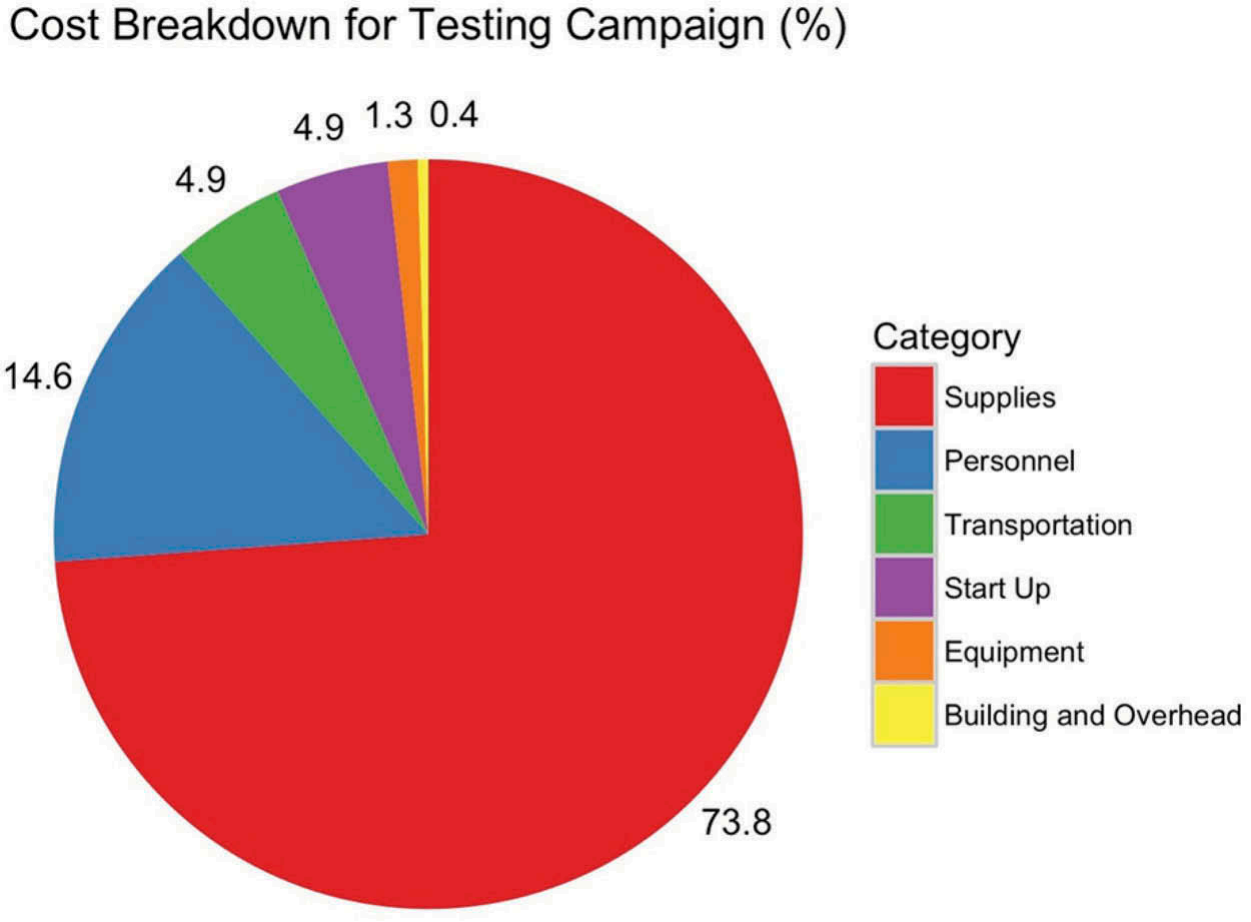
Costs by category for CHEWs HIV testing campaign.

The estimated programme cost was $132,167. Cost for programme components included supplies at $97,587 (73.8%), personnel at $19,348 (14.6%), transportation at $6,515 (4.9%), start-up costs of $6,421 (4.9%), equipment at $1,701 (1.3%), and building costs and overhead of $595 (0.4%) (Figure 4). The cost per test performed was $3.02, cost per positive test was $135.70, and cost per linkage was $212.15. Doubling the CHEWs stipend would increase the cost per test to $3.37, cost per positive test to $150.99, and cost per linkage to $236.06 (Supplementary File).

## Discussion

Lay community health extension workers (CHEWs) can be rapidly trained to scale-up home-based HIV testing and counselling (HTC) and linkage to care in a high-quality and low-cost manner to large numbers of people in a rural, high burden setting. Milat’s framework [10,11] provides a structured method for the programme implementers to reflect on which factors promoted scalability of this model of HIV counselling and testing and which require further modification. Understanding the successes and limitations of this programme is essential for ICOBI, the community-based organization that will continue to provide HIV care in this Ugandan community, and may be informative for other HIV care organizations who seek to optimize a home-based HTC programme for their region.

This programme effectively reached a large number of participants for HIV testing. With more than 43,000 persons tested, it was larger than all but four of the 39 home-based HIV testing studies included in a recent meta-analysis [7]. Furthermore, more than 47% of the people tested by CHEWs were men, which is a greater than the mean value of 40% reported for other community-based HIV testing studies [7]. Factors that may have contributed to the scale of this intervention were the relative density of this area, which facilitated travel by bicycle, and the highly motivated CHEWs, who were persons already living in these parishes and engaged as Village Health Team members.

The yield of this home-based HTC programme was 2.2% of persons testing positive for HIV, which is lower than the 5.1% of persons found to be HIV-positive through health facility testing during the same period. Despite the lower HIV positivity yield, home-based testing may still complement facility-based testing, because persons who received a test at home may not have otherwise sought testing at a health facility, or may have been identified at an earlier stage, before they developed clinical illness. Strategies to increase yield in future testing programmes may include the use of a screening tool to identify persons at greater risk of HIV infection, such as persons with an HIV-positive sexual partner [22]. Additionally, only 21.5% of people reported that this was their first HIV test, which is lower than the 30–50% of first-time testers found in recent studies in Uganda [23,24]. Prioritizing geographic areas which were not well represented in the first round of this programme may identify areas with a greater proportion of first-time testers. Geographic prioritization has been used previously to inform mobile HIV testing programmes [25].

As further evidence of programme effectiveness, nearly two-thirds of programme participants linked to care, which is an essential step towards achieving the second and third UNAIDS targets of initiating ART and achieving viral suppression. While fewer persons linked to care following HIV testing by CHEWs, this figure compares favourably with other studies of home-based HTC, which were conducted in a research setting [26,27]. Linkage to care could be further enhanced with additional follow-up by CHEWs to encourage clinic visits [7].

This programme achieved home-based HTC at low cost. The estimated cost per person tested was $3.02. A recent review of the costs of community-based testing found that home-based HCT costs ranged from $2.70 to $14.70 per person tested, whereas cost estimates for venue- and mobile-based approaches ranged from $8.30 to $42 [28]. While the variance in cost estimates depends on coverage achieved, HIV prevalence, and services offered (i.e., CD4 count), this programme is among the lowest cost HIV testing interventions reported. Despite the comparatively low HIV positivity rate (2.2%), the cost of identifying one HIV-positive person was $135.70, which is lower than most previously published estimates [28]. As government spending on health is estimated to be $9 US dollars per capita annually, this figure likely exceeds the current public funds available for HIV testing. Implementation of this programme in other settings would require sustained external support.

Several factors contributed to the low costs incurred in this intervention. First, the decentralized parish-level approach greatly reduced travel time and costs. Second, personnel expenses made up less than 15% of the cost. The $30 USD monthly stipend provided to CHEWs was below the range of figures reported in a recent review of lay counsellor work, which reported a range of $40 to $500 USD per month in other African countries [29].

The workforce resources required for this programme were modest. CHEWs did not have formal medical or paraprofessional training. They underwent a five-day training utilizing Ministry of Health materials, which could be delivered to others rapidly without needing adaptation. Employing lay people also contributed to the low cost of the intervention. The organizational resources provided by ICOBI may be more difficult to replicate in other settings. However, Uganda already has non-profit academic and community organizations that provide HIV services within a particular region, in addition to public sector services. Home-based HIV testing could be added to the portfolio of services offered by each organization.

Intervention fidelity to the CHEWs home-based HTC model was high. HIV testing proved to be an intervention that could be replicated accurately in this programme scale-up. The quality of testing was high, with agreement between results of the field-based rapid test and the lab-based ELISA on each of the more than 1800 samples that underwent confirmatory testing. In contrast to protocolized HIV testing, facilitating linkage to care requires employing motivational techniques, problem solving strategies to overcome obstacles, and sharing knowledge of the local clinical landscape that are specific to a local context [30]. These skills and strategies may need tailoring to a particular setting. Regarding other service delivery factors, this intervention could be combined with other community-based programmes to offer point-of-care testing for other medical conditions, such as diabetes or hypertension, which may make it additionally attractive to health programme planners [31]. Combining with other interventions may require a longer training period.

Contextual factors that likely contributed to the success of this programme were the engagement of local public health stakeholders and the respected role of ICOBI in the community. Programs that engage community health stakeholders have increased the probability of programme success [32–35]. ICOBI staff personally engaged with health officials at the village, district, and national levels to introduce the programme, and then invited all to celebrate the start of the programme with a programme launch celebration. Additionally, this well-established community group was successful at securing donor funding for this programme, which may not be accessible to other community groups. A plateau in the availability of donor funds for lay counsellor programmes raises concerns about the scalability and sustainability of these programmes unless they are incorporated into public sector budgets [29].

There were several limitations to this programme and its evaluation. First, the individual-level factors associated with HIV testing and care behaviours can best be assessed as part of a research study. Our programme evaluation could not assess the relationship between individual health behaviours and HIV testing, likelihood of testing positive, or linkage to care. Second, as personal identifiers were not collected, there may have been persons who enrolled in the testing programme more than once. However, there were not expected to be many duplicate enrolees, since CHEWs conducted the majority of testing in households. CHEWs were unlikely to accidently re-visit households in communities they knew well. Third, while Western blot testing was not available, guidelines support the use of a combination of rapid diagnostic tests and enzyme immune assays as reliable as conventional testing [36]. Finally, we did not enumerate households prior to testing and are not able to accurately estimate coverage, although there was high uptake overall.

Further investigations in this area should include additional examples of community-based lay counsellor HIV testing and counselling and linkage to care, with particular attention to strategies for finding persons at high risk for HIV acquisition. Additionally, community programmes should strive to engage local health stakeholders and harmonize testing protocols and counselling with public sector services. Cost-effectiveness analysis of these programmes may provide evidence for Ministries of Health to expand lay counsellor testing programmes, which could be critical for the sustainability of these programmes in an era of limited donor funding [29].

## Conclusions

Community directed programmes for lay counsellor home-based HIV testing and counselling can expand testing toward 90–90-90 targets at low cost, and could work synergistically with other testing approaches, such as testing partners of HIV-positive persons, outreach testing to men and young persons who are not routinely tested, and outreach to key populations at high risk of HIV acquisition, such as commercial sex workers and men who have sex with men. Systematic evaluation of programmes through routinely collected and analysed data can inform new programmes, as well as refine existing programmes. Opportunities to build and optimize such programmes should be supported.

## Abbreviations

ART, antiretroviral therapy; ICOBI, Integrated Community Based Initiatives; HTC: HIV testing and counselling

## Notes

To access the supplementary material to this article please see Supplementary Files under Article Tools online.
